# A Kinetic and Factorial Approach to Study the Effects of Temperature and Salinity on Growth and Toxin Production by the Dinoflagellate *Alexandrium ostenfeldii* from the Baltic Sea

**DOI:** 10.1371/journal.pone.0143021

**Published:** 2015-12-04

**Authors:** Pablo Salgado, José A. Vázquez, Pilar Riobó, José M. Franco, Rosa I. Figueroa, Anke Kremp, Isabel Bravo

**Affiliations:** 1 Departamento de Medio Ambiente, División de Investigación en Acuicultura, Instituto de Fomento Pesquero (IFOP), Punta Arenas, Chile; 2 Grupo de Reciclado y Valorización de Materiales Residuales (REVAL), Instituto de Investigaciones Marinas (IIM-CSIC), Vigo, Spain; 3 Instituto de Investigaciones Marinas (IIM-CSIC), Vigo, Spain; 4 Centro Oceanográfico de Vigo, Instituto Español de Oceanografía (IEO), Vigo, Spain; 5 Marine Research Centre, Finnish Environment Institute (SYKE), Helsinki, Finland; University of Connecticut, UNITED STATES

## Abstract

*Alexandrium ostenfeldii* is present in a wide variety of environments in coastal areas worldwide and is the only dinoflagellate known species that produces paralytic shellfish poisoning (PSP) toxins and two types of cyclic imines, spirolides (SPXs) and gymnodimines (GYMs). The increasing frequency of *A*. *ostenfeldii* blooms in the Baltic Sea has been attributed to the warming water in this region. To learn more about the optimal environmental conditions favoring the proliferation of *A*. *ostenfeldii* and its complex toxicity, the effects of temperature and salinity on the kinetics of both the growth and the net toxin production of this species were examined using a factorial design and a response-surface analysis (RSA). The results showed that the growth of Baltic *A*. *ostenfeldii* occurs over a wide range of temperatures and salinities (12.5–25.5°C and 5–21, respectively), with optimal growth conditions achieved at a temperature of 25.5°C and a salinity of 11.2. Together with the finding that a salinity > 21 was the only growth-limiting factor detected for this strain, this study provides important insights into the autecology and population distribution of this species in the Baltic Sea. The presence of PSP toxins, including gonyautoxin (GTX)-3, GTX-2, and saxitoxin (STX), and GYMs (GYM-A and GYM-B/-C analogues) was detected under all temperature and salinity conditions tested and in the majority of the cases was concomitant with both the exponential growth and stationary phases of the dinoflagellate’s growth cycle. Toxin concentrations were maximal at temperatures and salinities of 20.9°C and 17 for the GYM-A analogue and > 19°C and 15 for PSP toxins, respectively. The ecological implications of the optimal conditions for growth and toxin production of *A*. *ostenfeldii* in the Baltic Sea are discussed.

## Introduction

In recent decades, dinoflagellate species that are causative agents of harmful algal blooms (HABs) have been studied intensively, due to their global proliferation and their adverse effects on public health, recreation and tourism, fisheries, aquaculture and the ecosystems in which they are found. Although measures to counter HABs are still lacking, it is clear that the control of bloom events requires detailed knowledge of their basic features, including the adaptive strategies of the responsible dinoflagellate species and the environmental factors that regulate them [1]. Among toxin-producing dinoflagellates, members of the genus *Alexandrium* are the causative agents of the most widespread seafood poisoning syndrome caused by HABs, paralytic shellfish poisoning (PSP) syndrome. Toxigenic *Alexandrium* species are mainly distributed within coastal and temperate waters, although toxic populations are also found in sub-tropical, tropical, and perhaps even Arctic waters [2]. Currently, this genus includes 31 morphologically defined species, of which 12 produce PSP toxins [3, 4]. Among these well-known species, *Alexandrium ostenfeldii* (or synonym *Alexandrium peruvianum*) is the only member of the genus *Alexandrium* able to produce toxins of the cyclic imine type, including spirolide (SPX) and gymnodimine (GYM) [5–8]. Cyclic imine toxins are a family of structurally related marine neurotoxins of dinoflagellate origin that contaminate shellfish. Their basic structure consists of an imine moiety as a part of a bicyclic ring system [9]. These toxins display fast-acting toxicity when injected intraperitoneally in laboratory mice, although there have been no reported cases of poisoning in humans [10, 11]. The toxin profiles of *A*. *ostenfeldii* are complex. *A*. *ostenfeldii* was identified as the source of SPX toxins in Nova Scotia, Canada [5], even though it had been previously reported as a source of PSP-associated neurotoxin, which also causes a toxic syndrome [12]. In recent studies [6–8], GYM was shown to be produced by *A*. *ostenfeldii* strains of different geographic origin.

A recent study on phylogenetic, morphological, and toxin profiles of *A*. *ostenfeldii* and *A*. *peruvianum* strains from diverse geographic origins showed that *A*. *peruvianum* should be considered synonymous with *A*. *ostenfeldii*, and therefore discontinued as a distinct taxon [13]. Thus, the two species are considered synonymous herein. The first record of *A*. *ostenfeldii* was from the northern coast of Iceland [14], but the species has since been reported in most cold water environments, from the high latitudes of the Atlantic Ocean and northern Europe [12, 15–18] to the southern Pacific Ocean off the coast of austral Chile and Argentina [19–22]. However, *A*. *ostenfeldii* also occurs in warm waters, including off the coasts of Peru [23, 24], Malaysia [25], Spain [26], Italy [27], and Greece [28]. *A*. *ostenfeldii* also tolerates a wide range of salinities, based on its presence in the low-salinity environments of the Baltic Sea [29] and Chilean fiords and channels [22, 30] but also along the Mediterranean coast, where the salinities are higher [27, 31].

The toxin profiles of strains from those diverse environments also vary, with the production of SPX or PSP toxin by some strains depending upon the region of origin [6, 29]. For example, strains of *A*. *ostenfeldii* from the Baltic Sea mainly produce PSP toxins but not SPXs; those of the North Sea and Mediterranean Sea produce SPXs [6, 13]; and those of the Kattegat Sea (located between the Baltic Sea and the North Sea) produce both. The production of GYM toxins by Baltic Sea strains was recognized only recently [6, 8] and, thus far, only Narragansett and New River (USA) strains of *A*. *ostenfeldii* contain PSP toxins and the two cyclic imines (SPX and GYM) [7, 32, 33].

The relationship between environmental factors and toxin production by dinoflagellates is complex. Experimental studies have shown that the production of either SPXs or PSP toxins by *A*. *ostenfeldii* is influenced by salinity, temperature, and nutrients (see [29, 34, 35]). Whether this is also the case for the recently discovered GYM toxins in *A*. *ostenfeldii* strains is unclear. Thus, in the present work, we used a kinetic and factorial approach to study the effects of salinity and temperature on growth and toxin production by a strain of *A*. *ostenfeldii* isolated from the Baltic Sea.

## Material and Methods

### Culture conditions

*Alexandrium ostenfeldii* strain AOTV-B4A was isolated from the Baltic Sea (Åland, Finland) in summer 2004 and is maintained as part of our culture collection of toxic microalgae at the Spanish Institute of Oceanography in Vigo (CCVIEO: http://www.vgohab.es/). The cultures were acclimated gradually to different salinities (max. 3–4 salinity units at a time) and temperatures for at least during three transfers after reaching the stationary phase, according to the experimental conditions selected to develop the factorial design (Table 1). Pyrex glass bottles (1 L) containing 500 mL of L1 medium without silicate [36] were inoculated with exponentially growing cells (2,000–4,000 cells mL^-1^) to a final concentration of 900 cells mL^-1^. The medium was prepared using seawater collected from the Galician shelf at a depth of 5 m and adjusted to the salinities listed in Table 1 by the addition of sterile MQ water (Milli-Q; Millipore, USA). A photoperiod of 12 h of light (photon flux approximately 100 μmol m^–2^ s^–1^) and 12 h of darkness was used. Growth was monitored as cell yield (cells mL^-1^) throughout the growth cycle of *A*. *ostenfeldii*. Eight or nine samples (6–28 mL) were collected in total from each of the cultures during the experimental period and used in the toxin analyses (PSP toxins and cyclic imines: SPX and GYM) and cell counts. Sampling resulted in the removal of no more than 27% of the total volume of each culture. Samples used to determine cell counts were fixed with Lugol solution. Cell density was determined by light microscopy using a Sedgwick–Rafter chamber.

**Table 1:** Experimental domain and codification of the independent variables in the factorial rotatable design.

Coded values	Natural values	
	*S*	*T* (°C)
-1.41	5.0	12.5
-1	9.7	14.4
0	21.0	19.0
+1	32.3	23.6
+1.41	37.0	25.5

Codification: *V*_*c*_ = (*V*_*n*_ *-V*_*0*_)/*ΔV*_*n*._

Decodification: *V*_*n*_ = *V*_*0*_ + (*ΔV*_*n*_ × *V*_*c*_).

*Vn* = natural value of the variable to be codified.

*Vc* = codified value of the variable.

*V*_*0*_ = natural value in the center of the domain.

*ΔV*_*n*_ = increment of *V*_*n*_ for unit of *V*_*c*._

Harmful effects due to a high pH and the pH changes resulting from the different cell concentrations in the treatments were controlled through a pH-measurement experiment performed throughout the entire growth cycle of the *A*. *ostenfeldii* strain using two replicates at *S*5/*T*19 and *S*9.7/*T*23.6. The pH kinetics were very similar in the two cultures, which yielded high (*S*9.7/*T*23.6 maximum of 22,266 cells mL^-1^) and low (*S*5/*T*19 maximum of 8,485 cells mL^-1^) rates of growth. The pH varied during growth progression by 1 and 1.5 units, respectively, and never exceeded 9.10. The results showed that CO_2_ was not a limiting factor for cell growth in the cultures.

### Extraction and analysis of toxins

The content and relative proportions of PSP toxin and cyclic imines (SPXs and GYMs) in samples from cultures exposed to different experimental conditions were determined as follows: Two culture subsamples were filtered through GF/F glass-fiber filters (25 mm diameter; Whatman, Maidstone, England) and maintained in a freezer at –20°C. After two freeze/thaw cycles, the samples were sonicated (1 min, 50 Watts) and then centrifuged (14,000 rpm, 10 min, 5°C). One of the filters was extracted twice with 0.05 M acetic acid for PSP toxin analysis and the other twice with 100% methanol for SPX and GYM toxin analyses. The extracts (1.5 mL each) were kept at –20°C until used in the respective analysis, at which time they were left at ambient temperature and then filtered through 0.45-μm syringes filter.

PSP toxins were analyzed by high-performance liquid chromatography (HPLC) with post-column oxidation and fluorescence detection (FD) according to the method of Rourke et al. [37], with slight modifications, and by using a Zorbax Bonus RP (4.6 × 150 mm, 3.5 μm) column. SPX and GYM toxins were identified by liquid chromatography coupled to high-resolution mass spectrometry (LC–HRMS). The methanolic extracts were analyzed on a Dionex Ultimate 3000 LC system (Thermo Fisher Scientific, San Jose, California) coupled to an Exactive mass spectrometer (Thermo Fisher Scientific, Bremen, Germany) equipped with an Orbitrap mass analyzer and a heated electrospray source (H–ESI II). Nitrogen (purity > 99.999%) was used as the sheath gas, auxiliary gas, and collision gas. The instrument was calibrated daily in positive and negative ion modes. Mass acquisition was performed in positive ion mode without and with all ion fragmentation (AIF) higher-energy collisional dissociation (HCD) of 45 eV. The mass range was *m/z* 100–1000 in both full-scan and AIF modes. SPXs and GYMs were separated and quantified according to the Standardized Operating Procedure validated by the European Union Reference Laboratory for Marine Biotoxins [38]. In case another SPX or GYM different from the standards was identified in samples, it was quantified as 13-desmethyl SPX-C or as GYM-A equivalents, based on the respective calibrations available and assuming equal responses. The X-Bridge C18 (100 × 2.1 mm, 2.5 μm) column was maintained at 25°C; the injection volume was 20 μL and the flow rate 400 μL min^–1^ (for details, see [6]).

### Mathematical modeling of *A*. *ostenfeldii* growth and toxin production

The sigmoid kinetics of *A*. *ostenfeldii* growing under different experimental conditions were fitted to the logistic equation [39, 40]:

$$G=\frac{G_{m}}{1+\text{exp}\left[2+\frac{4v_{m}}{G_{m}}\left(\lambda -t\right)\right]}$$ (1)

This equation can be easily reformulated to obtain parameters that describe and characterize the different phases represented in the sigmoid growth curves [41]:

$$G=\frac{G_{m}}{1+\text{exp}\left[\;\mu_{m}\left(\tau -t\right)\right]}\;\text{and}\;t_{m}=\tau +\frac{G_{m}}{2v_{m}}$$ (2)

where *G* is the dinoflagellate growth concentration (cells mL^-1^); *t*, the culture time in days (d); *G*_*m*_, the maximum cell concentration (cells mL^-1^); *μ*_*m*_, the maximum specific growth rate (d^−1^); *τ*, the time required to achieve the semi-maximum cell concentration or *Gm*/2 (d); *v*_*m*_, the maximum growth rate (cells mL^−1^ d^−1^); *λ*, the lag phase (d); and *t*_*m*_, is the time required to achieve the beginning of *G*_*m*_ or plateau phase (d).

Net toxin production followed first-order kinetics [39], described by:

$$T_{x}=T_{0}\text{exp}\;\left(rt\right)$$ (3)

where *T*_*x*_ is the net production of the PSP (pg cell^-1^) or the GYM-A analogue (pg GYM-A eq. cell^-1^) toxins; *t*, the culture time (d); *T*_*0*_, the initial toxin content (pg cell^-1^); and *r*, the specific net toxin production rate (d^−1^).

### Numerical methods for growth and toxin production curve modeling

Dinoflagellate growth and net toxin production were modeled by minimizing the sum of the quadratic differences between the observed and predicted values, using the non-linear least-squares (quasi-Newton) method provided by the macro “Solver” of the Microsoft Excel spreadsheet. Confidence intervals from the parametric estimates (Student’s *t* test) and the consistency of the mathematical models (Fisher’s *F* test) and residual analysis (Durbin-Watson test) were evaluated by “SolverAid” macro (Levie's Excellaneous website: http://www.bowdoin.edu/~rdelevie/excellaneous).

### Experimental design and statistical analysis

The effects of the independent variables salinity (*S*), in the range 5–37, and temperature (*T*), in the range 12.5–25.5°C, on the kinetic parameters that characterize the growth (*G*, in cells mL^-1^) and net toxin production (*T*_*x*_, in pg cell^-1^) of *A*. *ostenfeldii* were studied using a rotatable second-order design, with five replicates in the center of the experimental domain [42]. Table 1 summarizes the encoding of the independent variables and the experimental conditions employed.

Orthogonal least-squares calculation of the factorial design data were used to obtain [42] empirical equations describing the combined effects of the environmental factors (*S* and *T*) on the kinetic parameters obtained from Eqs (1–3). The general form of the polynomial equations is:

$$R=b_{0}+\sum_{i=1}^{n}b_{i}X_{i}+\sum_{\begin{array}{l} i=1\\ j>i \end{array}}^{n-1}\sum_{j=2}^{n}b_{ij}X_{i}X_{j}+\sum_{i=1}^{n}b_{ii}X_{i}^{2}$$ (4)

where *R* represents the response (dependent variable) to be modeled (growth and net toxin production parameters); *b*_*0*_ is the constant coefficient; *b*_*i*_, the coefficient of the linear effect; *b*_*ij*_, the coefficient of the interaction effect; *b*_*ii*_, the coefficients of the squared effect; *n*, the number of variables; and *X*_*i*_ and *X*_*j*_, the independent variables (*S* and *T*). The statistical significance of the coefficients was verified using Student t-test (α = 0.05). Goodness-of-fit was established as the adjusted determination coefficient (*R* ^*2*^_*adj*_), and the model’s consistency by Fisher’s *F* test (α = 0.05) using the following mean squares ratios:

**The model is acceptable when**

*F1* = Model / Total error                                   ${F1\;\geq F}_{den}^{num}$

*F2* = (Model + Lack of fit) / Model                 ${F2\;\leq F}_{den}^{num}$

*F3* = Total error / Experimental error        ${F3\;\leq F}_{den}^{num}$

*F4* = Lack of fit / Experimental error                ${F4\;\leq F}_{den}^{num}$

where $F_{den}^{num}$ are the theoretical values for α = 0.05, with the corresponding degrees of freedom for numerator (num) and denominator (den). The model is acceptable when *F1* and *F2* are validated. *F3* and *F4* were additionally calculated to improve the degree of robustness and the consistency of the empirical equations obtained. All fitting procedures, coefficient estimates, and statistical calculations were performed on a Microsoft Excel spreadsheet.

## Results

### Toxin characterization

The LC analyses showed detectable amounts of PSP toxins in all of the culture extracts of *A*. *ostenfeldii* strain AOTV-B4A. The toxin profile was dominated by gonyautoxin (GTX)-3, followed by saxitoxin (STX), and GTX-2 in proportions of 62.7%, 35.5%, and 1.8%, respectively. The proportions were similar in all cultures. In LC–HRMS analyses of the culture methanolic extracts, GYM-A and GYM-B/-C analogues but not SPXs were detected. Since only the standard for GYM-A is available, an equimolar response for the GYM-A analogue detected was assumed and the analogue was quantified as GYM-A equivalents (pg GYM-A eq. cell^-1^). In addition, although all culture samples were also screened for the presence of 12-methyl GYM, it was not detected under any conditions.

### Growth and toxin production kinetics

**Fig 1:**
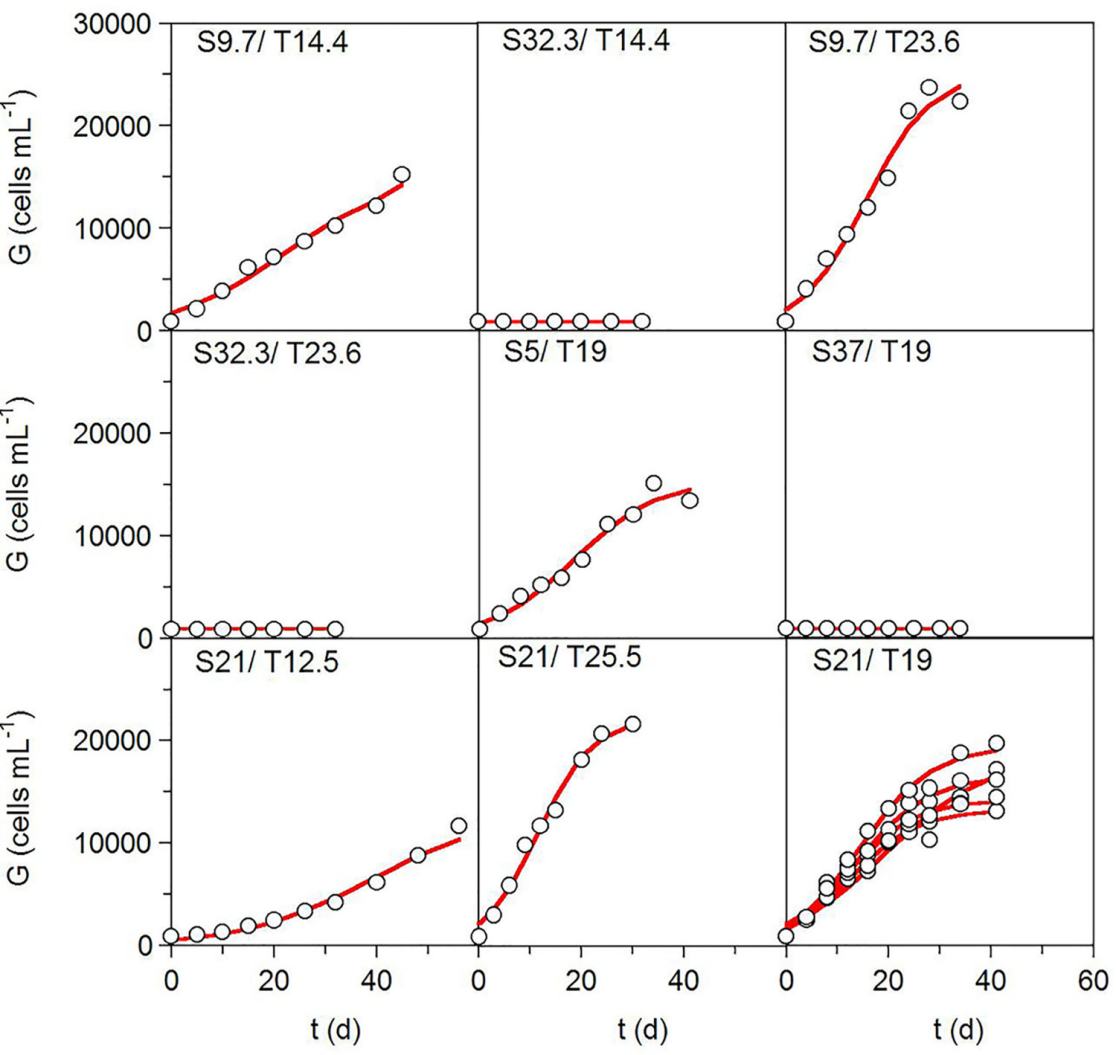
Growth kinetic profiles. Growth kinetics of *Alexandrium ostenfeldii* strain AOTV-B4A cultivated under the environmental conditions defined by the factorial design summarized in Table 1. Experimental data (symbols) were fitted to Eq (1) (lines).

Both the analysis of the growth of *A*. *ostenfeldii* under the conditions defined by the factorial design (Table 1) and the kinetic profiles fitted to the experimental data according to Eq (1) are shown in Fig 1. Table 2 lists the values of the kinetic parameters and provides the data used in the statistical analyses of the numerical fittings. The predictive ability of Eq (1) in modeling the experimental data was high, as shown by determination coefficients (R^2^) ≥ 0.965. All of the parameters for *A*. *ostenfeldii* growth, except the numerical values of the lag phases (*λ*), were statistically significant (α = 0.05). Autocorrelation was not observed in the residuals distribution (data not shown). In three of the experimental conditions (salinities ≥ 32), there was no significant growth of *A*. *ostenfeldii*, as determined by the kinetic analysis; the cell yields were not higher than the inoculum (900 cells mL^-1^) (Fig 1). In those cases, the values of the parameters used as dependent variables (responses) in the subsequent response-surface analysis (RSA) and calculation were set at zero.

**Table 2:** Summary of the parameter values (dependent variables) obtained from fitting the data on *A*. *ostenfeldii* growth to Eqs (1) and (2). *X_1_*: salinity and *X_2_*: temperature (°C). The natural values of the experimental conditions are shown in brackets.

Independent variables		Growth parameters							
*X*_*1*_: *S*	*X*_*2*_: *T*	*G*_*m*_ (cells mL^-1^)	*v*_*m*_ (cells mL^-1^ d^-1^)	*λ* (d)	*μ*_*m*_ (d^-1^)	*τ* (d)	*t*_*m*_ (d)	R^2^	p-value
-1 (9.7)	-1 (14.4)	17,912 ± 6,775	351.2 ± 88.6	1.0 (NS)	0.082 ± 0.043	25.5 ± 12.3	50.0 ± 24.6	0.976	<0.001
1 (32.3)	-1 (14.4)	NGD	NGD	NGD	NGD	NGD	NGD	NGD	NGD
-1 (9.7)	1 (23.6)	25,239 ± 6,775	983.1 ± 314.4	2.8 (NS)	0.156 ± 0.071	15.7 ± 4.2	28.5 ± 9.2	0.973	<0.001
1 (32.3)	1 (23.6)	NGD	NGD	NGD	NGD	NGD	NGD	NGD	NGD
-1.41 (5)	0 (19)	15,372 ± 3,132	479.3 ± 141.4	2.3 (NS)	0.125 ± 0.054	18.3 ± 4.9	34.4 ± 11.0	0.97	<0.001
1.41 (37)	0 (19)	NGD	NGD	NGD	NGD	NGD	NGD	NGD	NGD
0 (21)	-1.41 (12.5)	13,110 ± 2,389	261.1 ± 52.0	14.1 ± 5.0	0.080 ± 0.024	39.2 ± 6.7	64.3 ± 13.4	0.982	<0.001
0 (21)	1.41 (25.5)	22,237 ± 2,772	1,053.5 ± 257.8	1.1 (NS)	0.190 ± 0.060	11.7 ± 2.2	22.3 ± 5.0	0.987	<0.001
0 (21)	0 (19)	18,361 ± 3,542	476.8 ± 94.9	0.4 (NS)	0.104 ± 0.034	19.6 ± 5.0	38.9 ± 10.7	0.983	<0.001
0 (21)	0 (19)	16,523 ± 1,506	608.5 ± 125.3	0.5 (NS)	0.147 ± 0.038	14.0 ± 2.1	27.6 ± 5.0	0.988	<0.001
0 (21)	0 (19)	13,222 ± 1,838	512.7 ± 184.2	-0.4 (NS)	0.155 ± 0.068	12.5 ± 3.3	25.4 ± 7.8	0.965	<0.001
0 (21)	0 (19)	14,234 ± 1,435	538.4 ± 141.5	-1.2 (NS)	0.151 ± 0.049	12.0 ± 2.4	25.2 ± 5.8	0.981	<0.001
0 (21)	0 (19)	19,459 ± 2,062	710.2 ± 161.8	-1.1 (NS)	0.146 ± 0.042	14.8 ± 2.5	28.4 ± 5.7	0.986	<0.001

Codification: V_**c**_ = (V_n_–V_0_)/ ΔV_n_; Decodification: V_n_ = V_0_+(ΔV_n_×V_c_)

V_n_ = natural value in the center of the variable to be codified; ΔV_n_ = increment of V_n_ per unit of V_c_. V_c_ = codified value of the variable; V_0_ = natural value in the center of the domain

NS: not significant; NGD: no growth detected. Error values associated with the parameter determinations are the confidence intervals (CI) for α = 0.05.

**Fig 2:**
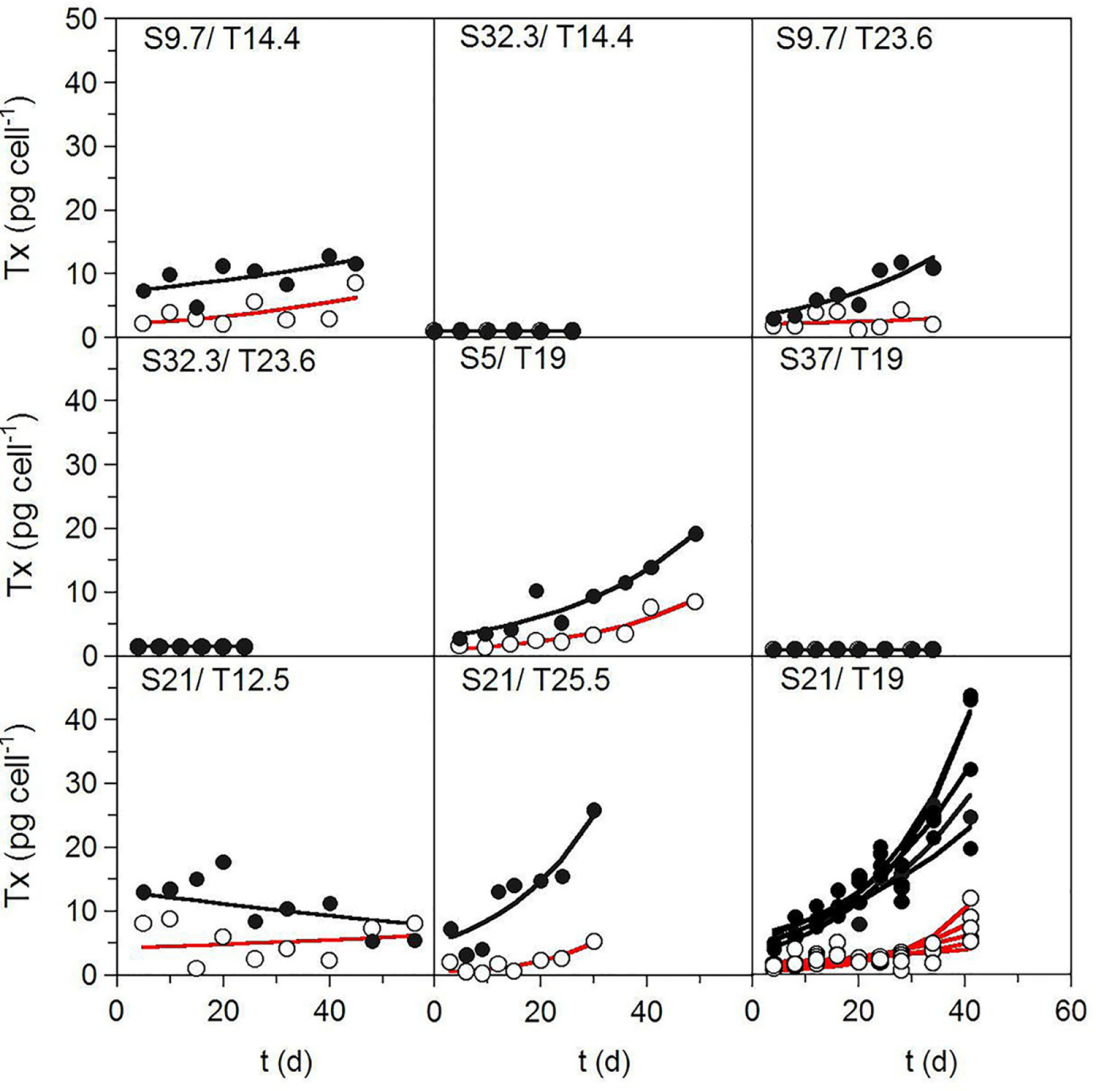
Toxin-production kinetic profiles. Kinetics of net toxin production by strain AOTV-B4A cultivated under the environmental conditions defined by the factorial design summarized in Table 1. ○: PSP toxins (pg cell^-1^), ●: GYM-A analogue (pg GYM-A eq cell^-1^). Experimental data (symbols) were fitted to Eq (3) (lines).

Fig 2 shows the kinetics of the net production of the PSP toxins (sum of GTX-3, GTX-2, and STX) and the GYM-A analogue under the conditions defined by the factorial design (Table 1) and fitted to the first-order kinetic model (3). In general, the experimental kinetics of the net production of those biotoxins over time were acceptably modeled by Eq (3). The coefficients of determination of the fittings were in the range of 0.246–0.957 for PSP toxins and 0.101–0.981 for GYM-A analogue. The kinetics of both groups of toxins were consistent with a mixed-growth-associated model, since content toxin increased during the exponential phase of growth and continued to increase during stationary phase (Figs 1 and 2). No change in toxin content was detected under the experimental conditions in which there was no significant growth (salinities ≥ 32) (Fig 2).

### Combined effect of temperature and salinity on growth parameters by RSA

The simultaneous effects of the environmental factors *S* and *T* on the kinetic parameters of *A*. *ostenfeldii* obtained from the logistic model (Table 2) were studied using a RSA. For the cases in which no growth detected (NGD in Table 2), the experimental response was considered to be zero. The design and numerical responses of the 2-factor rotatable design are summarized in Table 2. Parameter data describing the growth of *A*. *ostenfeldii* were converted into second-order polynomial equations as a function of *S* and *T*. The polynomial model describing the correlation between the responses and the variables therefore followed the general form described by Eq (4) (Table 3).

**Table 3:** Second-order equations describing the effects of *S* and *T* on the growth parameters of *A*. *ostenfeldii* AOTV-B4A (used in coded values according to the criteria defined in Table 1). The coefficient of adjusted determination (*R* ^*2*^_*adj*_) and the *F*-values (*F*_*1*_, *F*_*2*_, *F*_*3*_ and *F*_*4*_) are also shown. S: significant; NS: non-significant.

Parameters	*G*_*m*_ (cells mL^-1^)	*v*_*m*_ (cells mL^-1^ d^-1^)	*μ*_*m*_ (d^-1^)	*τ* (d)	*t*_*m*_ (d)
***b***_***0***_ **(intercept)**	16,365	569.6	0.141	14.60	29.14
***b***_***1***_ **(*S*)**	-8,037	-252.0	-0.052	-8.40	-15.92
***b***_***2***_ **(*T*)**	2,623	219.3	0.029	-6.09	-10.12
***b***_***12***_ **(*S***x***T*)**	NS	-158.0	NS	NS	NS
***b***_***11***_ **(*S*** ^***2***^ **)**	-4,881	-194.7	-0.049	-4.50	-8.68
***b***_***22***_ **(*T*** ^***2***^ **)**	NS	NS	NS	3.69	NS
***R*** ^***2***^_***adj***_	0.829	0.844	0.773	0.689	0.703
***F1***	20.33	17.17	14.63	7.65	10.46
	$[F_{9}^{3}$ = 3.86] ⇒ *S*	$[F_{8}^{4}$ = 3.84] ⇒ *S*	$[F_{9}^{3}$ = 3.86] ⇒ *S*	$[F_{8}^{4}$ = 3.84] ⇒ *S*	$[F_{9}^{3}$ = 3.86] ⇒ *S*
***F2***	0.416	0.545	0.438	0.614	0.469
	$[F_{3}^{8}$ = 8.85] ⇒ *S*	$[F_{4}^{8}$ = 6.04] ⇒ *S*	$[F_{3}^{8}$ = 8.85] ⇒ *S*	$[F_{4}^{8}$ = 6.04] ⇒ *S*	$[F_{3}^{8}$ = 8.85] ⇒ *S*
***F3***	1.727	2.15	20.52	3.98	3.46
	$[F_{4}^{9}$ = 5.99] ⇒ *S*	$[F_{4}^{8}$ = 6.04] ⇒ *S*	$[F_{4}^{9}$ = 5.99] ⇒ *S*	$[F_{4}^{8}$ = 6.04] ⇒ *S*	$[F_{4}^{9}$ = 5.99] ⇒ *S*
***F4***	2.308	3.29	20.52	6.96	5.44
	$[F_{4}^{5}$ = 6.26] ⇒ *S*	$[F_{4}^{4}$ = 6.39] ⇒ *S*	$[F_{4}^{5}$ = 6.26] ⇒ *S*	$[F_{4}^{4}$ = 6.39]⇒ *NS*	$[F_{4}^{5}$ = 6.26] ⇒ *S*

A high proportion of variability (83% for *G_m_* and 84% for *v_m_*) was successfully explained by the second-order equations. The agreement between the experimental and predicted data was always > 69% (Table 3). The robustness of the equations was perfect in all cases except the *F4* (Fisher F test) of *τ*, which was not significant. Thus, the empirical equations shown in Table 3 were very good predictors of the growth of *A*. *ostenfeldii* in the *S* and *T* ranges evaluated in this study.

**Fig 3:**
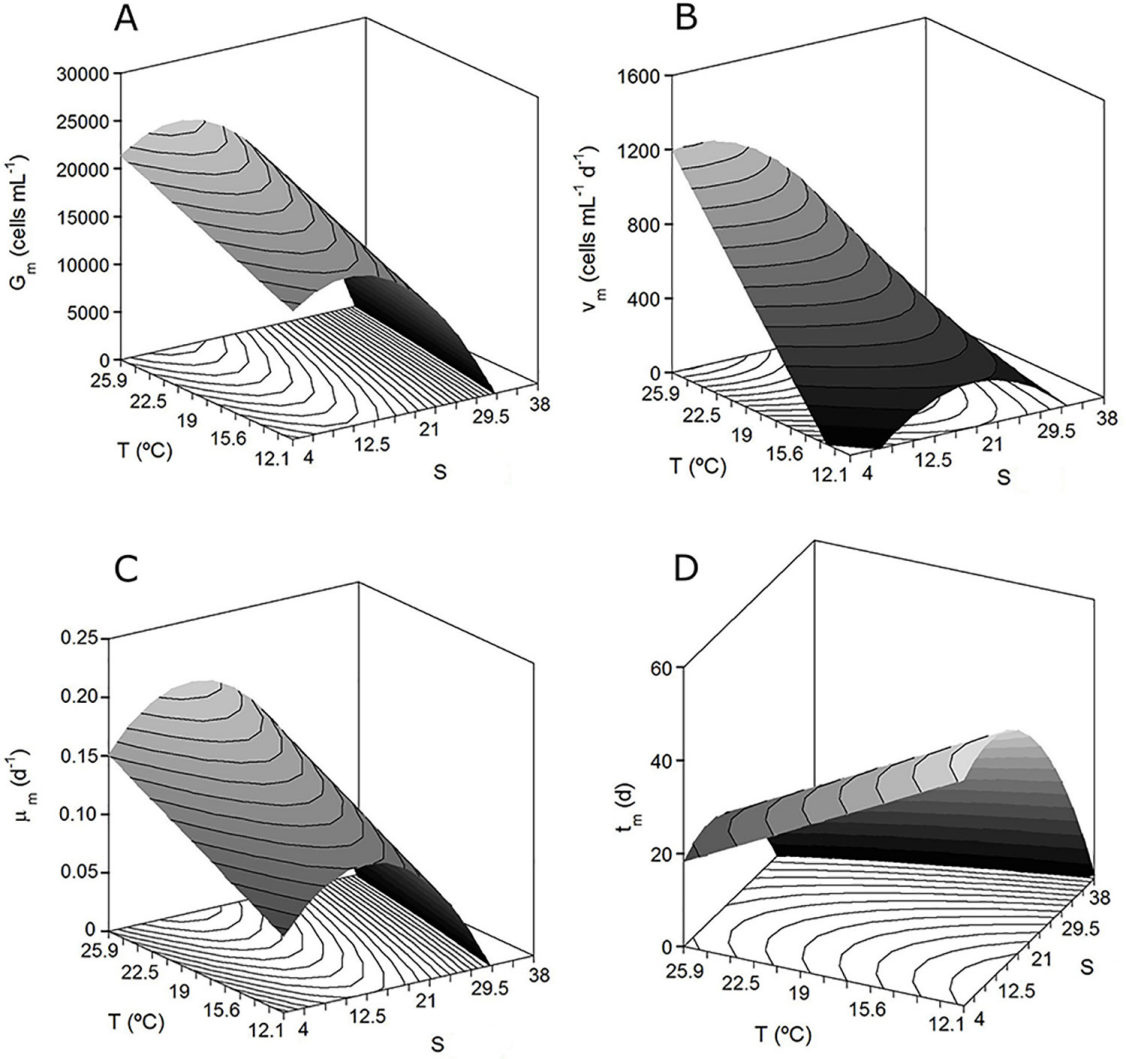
Combined effects of temperature and salinity on growth by RSA. Theoretical response surfaces describing the combined effects of temperature and salinity on the kinetic parameters described by Eqs (1) and (2): (A) maximum growth (*G*_*m*_), (B) maximum growth rate (*v*_*m*_), (C) specific maximum growth rate (*μ*_*m*_), and (D) time to achieve the plateau phase (*t*_*m*_).

Fig 3 shows the theoretical surfaces for each parameter as the response of a dependent variable. The results of the multivariate analysis showed that, for all growth parameters, the effect of *T* was only linear whereas *S* always had significant quadratic negative terms (*P* < 0.05). The coefficient of interaction between the variables (*S* × *T*) was only significant for the *v_m_* response. The statistical significance of the coefficient of *S* ^*2*^ and its negative value were graphically translated as a convex dome with a clear maximum point within the experimental domain of the salinity (Fig 3). The salinity concentrations that maximize *A*. *ostenfeldii* growth were determined by mathematical optimization using the numerical or manual derivation of the equations in Table 3 [43]. The natural values of those optima and the maximum value of the responses in each case (*Y_max_*) are summarized in Table 4. Thus, the conditions yielding the maximum growth of the dinoflagellate (average of the *G_m_*, *v_m_* and *μ_m_* results) were a salinity of 11.2 and a temperature of at least 25.5°C.

**Table 4:** Optimal values of salinity and temperature (*S*_*opt*_ and *T*_*opt*_) needed to obtain the maximum values (*Y*_*max*_) using the equations shown in Table 3 and for the different dependent variables studied (growth parameters).

	*G*_*m*_ (cells mL^-1^)	*v*_*m*_ (cells mL^-1^ d^-1^)	*μ*_*m*_ (d^-1^)	*τ* (d)	*t*_*m*_ (d)
***S***_***opt***_	11.68	6.79	15.04	10.44	10.63
***T***_***opt***_	>25.5	>25.5	>25.5	<12.5	<12.5
***Y***_***max***_	23,607	1,206	0.198	17.69	51.62

### Combined effect of temperature and salinity on toxin production by RSA

**Fig 4:**
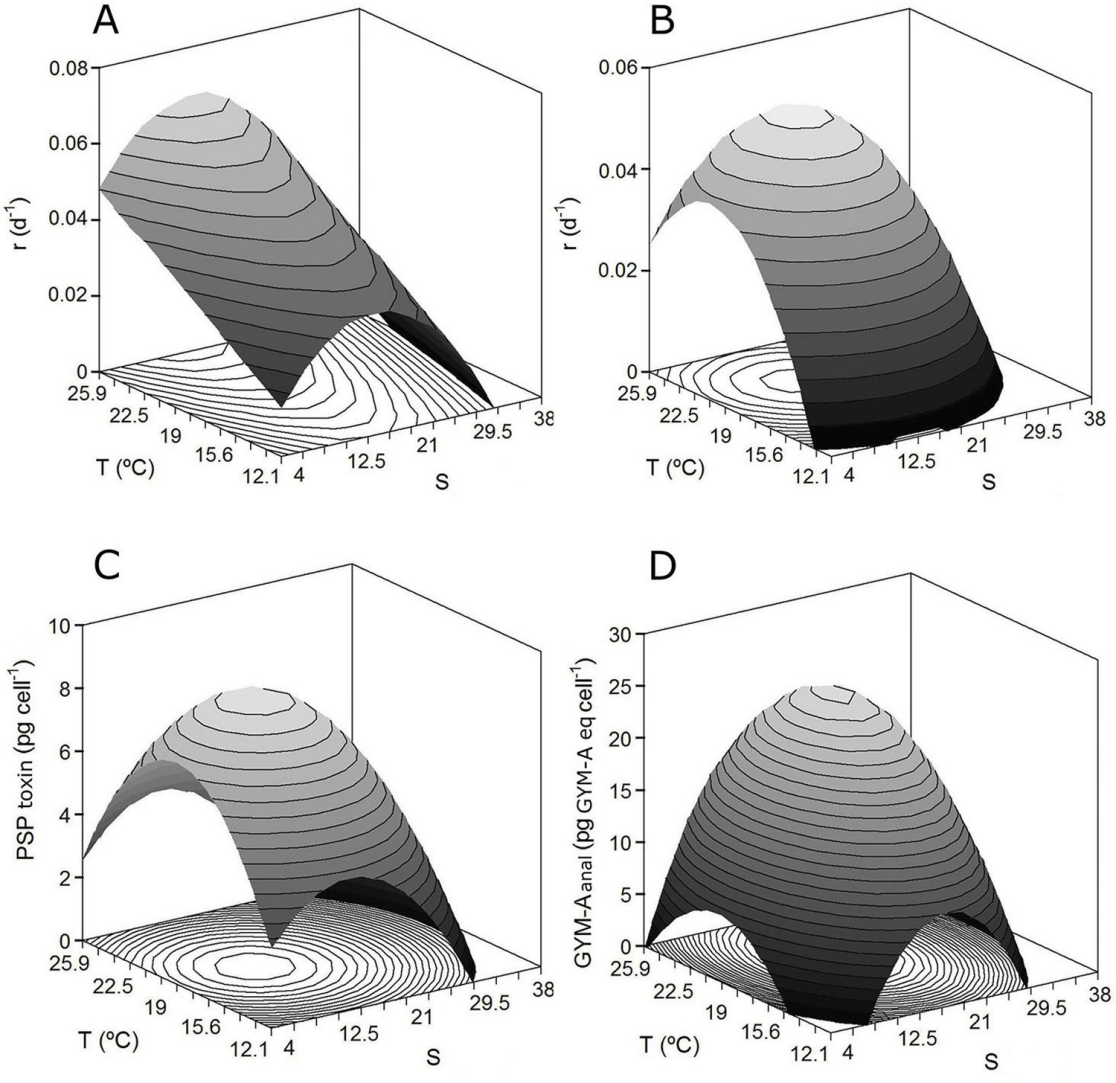
Combined effects of temperature and salinity on toxin production by RSA. Theoretical response surfaces describing the combined effects of temperature and salinity on the specific (A) PSP toxin and (B) GYM-A analogue net production rates (*r*), and on the net production of (C) PSP toxins (pg cell^-1^) and (D) GYM-A analogue (pg GYM-A eq. cell^-1^) at the end of the *A*. *ostenfeldii* culture period.

Based on Eq (3), the specific net production rates (*r*) of the PSP toxins and the GYM-A analogue were selected as the response to be studied with respect to *T* and *S*. The effects of temperature and salinity on the toxin concentrations obtained at the end of their production by *A*. *ostenfeldii* cells were also evaluated. Fig 4 depicts the surfaces predicted by the second-order equations shown in Table 5. In all cases, the coefficient of the salinity effect (linear and quadratic) was negative, with a parabolic pattern (dome surface), and was in complete concordance with the results obtained for dinoflagellate cell growth. The influence of *T* was also quadratic except for the *r*-response of PSP toxin, in which only a linear term was significant as previously described for the growth of *A*. *ostenfeldii* (Fig 3).

**Table 5:** Second-order equations describing the effects of *T* and *S* on net toxin productions (PSP toxin and GYM-A analogue) by *A*. *ostenfeldii*. The coefficient of adjusted determination (*R* ^*2*^_*adj*_) and the *F*-values (*F*_*1*_, *F*_*2*_, *F*_*3*,_ and *F*_*4*_) are also shown. S: significant; NS: non-significant.

	PSP toxin		GYM-A analogue	
Parameters	*r* (d^-1^)	PSP toxin (pg cell^-1^)	*r* (d^-1^)	GYM-A analogue (pg GYM-A eq. cell^-1^)
***b***_***0***_ **(intercept)**	0.047	7.82	0.047	24.57
***b***_***1***_ **(*S*)**	-0.014	-2.85	-0.015	-4.47
***b***_***2***_ **(*T*)**	0.012	NS	0.013	3.98
***b***_***12***_ **(*S***x***T*)**	NS	NS	NS	NS
***b***_***11***_ **(*S*** ^***2***^ **)**	-0.017	-2.24	-0.015	-10.91
***b***_***22***_ **(*T*** ^***2***^ **)**	NS	-2.00	-0.013	-5.95
**(*R*** ^***2***^_***adj***_ **)**	0.504	0.664	0.715	0.819
***F1***	4.05	8.91	8.54	14.53
	$[F_{9}^{3}$ = 3.86] ⇒ *S*	$[F_{9}^{3}$ = 3.86] ⇒ *S*	$[F_{8}^{4}$ = 3.84] ⇒ *S*	$[F_{8}^{4}$ = 3.84] ⇒ *S*
***F2***	0.713	0.465	0.571	0.56
	$[F_{3}^{8}$ = 8.85] ⇒ *S*	$[F_{3}^{8}$ = 8.85] ⇒ *S*	$[F_{4}^{8}$ = 6.04] ⇒ *S*	$[F_{4}^{8}$ = 6.04] ⇒ *S*
***F3***	5.370	1.537	1.265	5.94
	$[F_{4}^{9}$ = 5.99] ⇒ *S*	$[F_{4}^{9}$ = 5.99] ⇒ *S*	$[F_{4}^{8}$ = 6.04] ⇒ *S*	$[F_{4}^{8}$ = 6.04] ⇒ *S*
***F4***	8.867	1.967	1.530	10.88
	$[F_{4}^{5}$ = 6.26] ⇒ *NS*	$[F_{4}^{5}$ = 6.26] ⇒ *S*	$[F_{4}^{4}$ = 6.39] ⇒ *S*	$[F_{4}^{4}$ = 6.39] ⇒ *NS*

The optimal *S* and *T* values needed to maximize toxin concentrations and the theoretical maximum values of the variables (*Y_max_*) calculated from these optima are summarized in Table 6. These levels were different depending on the toxin; thus, the *S_opt_* values for PSP toxin and GYM-A analogue were 15 and 17, respectively (calculated in each case as the average of the two responses selected). The *T_opt_* for the GYM-A analogue was 20.9°C whereas that for PSP toxin was 19°C.

**Table 6:** Optimal values of salinity and temperature (*S*_*opt*_ and *T*_*opt*_) needed to obtain the maximum values (*Y*_*max*_) using the equations shown in Table 5 and for the different dependent variables studied (toxin productions).

	PSP toxin		GYM-A analogue	
	*r* (d^-1^)	PSP toxin (pg cell^-1^)	*r* (d^-1^)	GYM-A analogue (pg GYM-A eq. cell^-1^)
***S***_***opt***_	16.1	13.8	15.3	18.7
***T***_***opt***_	>25	19.0	21.2	20.5
***Y***_***max***_	0.067	8.73	0.054	25.7

## Discussion

### Growth and toxin production kinetics and the combined effects of temperature and salinity

The results of the present work clearly demonstrated the effects of temperature and salinity on the growth of one strain of *A*. *ostenfeldii* from the Baltic Sea. The values of the six kinetic parameters used (*G_m_*, *v_m_*, *λ*, *μ_m_*, *τ*, and *t_m_*) during each experimental condition were accurately predicted by the logistic function of Eq (1). The resulting sigmoid patterns and the validity of the model (1) that described them have been frequently reported for microorganisms cultivated under batch conditions [44–49] and for higher organisms subjected to extensive or intensive feeding [50–52]. The growth of marine organisms, including bacteria, rotifers, molluscs, dinoflagellates, and microalgae, is also well-fitted by a logistic function, reflecting the involvement of autocatalytic pathways [53–57]. Our results are in agreement with the growth rates reported for strains of *A*. *ostenfeldii* isolated from the Baltic Sea, Skagerrak, North Sea, and Nova Scotia [29, 34, 58] and for the yessotoxin-producing dinoflagellate *P*. *reticulatum* [39]. However, the values of the parameters *G_m_* or *K*, *v_m_* and *μ_m_* were generally lower for *A*. *ostenfeldii* than for *P*. *reticulatum*. Also, the growth of *A*. *ostenfeldii* was slower than that of *Alexandrium* species (*A*. *tamarense*, *A*. *minutum*, and *A*. *tamutum*) in Scottish waters [59] and *Alexandrium* isolates from other latitudes [60–62].

RSA based on a factorial design is used to optimize the effect of environmental factors for a small number of experiments (13, with 5 replicates in the center of the experimental domain for a second-order rotatable design) [42]. The empirical equation obtained from this analysis provides the information needed to predict the values of the dependent variables in the experimental range studied. Although this type of approach is not commonly used to study the growth of marine organisms in response to different effectors, previous results have demonstrated its validity and its potential as a process predictor. For example, the influence of three factors (temperature, salinity, and irradiance) on the growth kinetics of *P*. *reticulatum* was studied by means of a first-order factorial design [39]. Similar statistical tools were used to assess the positive and negative effects of salinity, inoculum size, and temperature on cyst and planozygote formation by *A*. *minutum* [63]. Analysis of the enhancement of rotifer (*Brachionus plicatilis*) growth in the presence of a combination of lactic acid bacteria was optimized by RSA [55]. The approach used in this work can be applied to define the environmental windows allowing species growth, especially those species with an extended geographic distribution, as is the case for *A*. *ostenfeldii*. Although generalizations based on culture studies should consider within-species variation [64, 65], it adds to the limited data available on the autecology of this species and allows conclusions to be drawn regarding the biogeography of the different populations of this dinoflagellate, based on comparisons between culture data and data on the environmental conditions of its habitats.

In our study, the temperature- and salinity-dependent growth window for Baltic *A*. *ostenfeldii* AOTV-B4A reflected the broad tolerance of this strain to a wide range of temperatures and to low salinities. In fact, the only condition completely limiting growth was a salinity ≥ 32. Although we could not determine the maximum threshold value for salinity, in a previous study it was 25 [6]. In the present study, cell growth occurred at a salinity range of 5–21 and at temperatures of 12.5–25.5°C, with optima at a salinity of 11.2 and a minimum temperature of 25.5°C. These results, and particularly the optimal salinity, are consistent with the values reported by Kremp et al. [16] for *A*. *ostenfeldii* strains AOTV-A1 and AOTV-A4, isolated, as our strain, from the Åland islands (Finnish Baltic Sea). Similar temperature ranges for growth were determined by Østergaard and Moestrup [17] for cultures of a Danish strain isolated from Limfjord (Kattegat Sea), whereas salinities allowing growth were between 10 and 40, with optimum growth achieved at 15–20. We have no information on the salinities of the waters where the Danish strain was isolated; however, its adaptation to a higher salinity suggests that it is from an area of the Kattegat Sea located closer to the North Sea than to the most northern part of the Baltic Sea [29], which is where our strain was obtained. The positive effects of temperature on *G_m_*, *v_m_*, and *μ_m_* (increasing response at increasing temperature) are compatible with the Arrhenius theory but with a linear rather than the typical exponential relationship. This may be due to the narrow experimental range selected in this study and/or to the type of factorial design, in which only linear and quadratic coefficients, and not exponential expressions, could be evaluated. The robustness of our results was confirmed by the negative value of the temperature linear coefficient in the equations describing *τ* ant *t_m_* (Table 3), which were inversely proportional to those obtained for the other parameters. This was expected because when growth is higher and faster (high *G_m_* and high *v_m_* and *μ_m_*), the values of *τ* and *t_m_* are smaller.

Our results also demonstrated the influence of temperature and salinity on the kinetics of net toxin production of *A*. *ostenfeldii*. The bioproduction of lipophilic (GYM-A analogue) and PSP toxins in most cases occurred concomitantly during the exponential growth and stationary phases, following characteristics of mixed metabolites as described by the Luedeking-Piret definition (Figs 1 and 2). A similar behavior was reported for yessotoxin production by *P*. *reticulatum*, nodularin synthesis by *Nodularia spumigena* [54], and net PSP production by *A*. *ostenfeldii* from the Baltic Sea [29] and by several other *Alexandrium* species from diverse geographic origins [61, 66, 67]. Nevertheless, in our study there were also culture conditions in which toxin production differed from that described above. Specifically, when the cultures were exposed to low temperature, net toxin production was not consistent with growth (see plots for 12.5°C in Figs 1 and 2), suggesting the involvement of other factors. In fact, in our experiment, in the absence of a clear stationary phases (temperatures of 12.5°C and 14.4°C), the error associated with the *G_m_*-parameter was higher than in cultures in which a well-defined asymptotic phase occurred. The changes in cell size may explain this difference. Both Granéli and Flynn [68] and Anderson et al. [69] noted that, during the growth cycle, the observed changes in the culture and in cell toxin content may simply reflect changes in cell size and the variability of growth or life-cycle stages as a function of changes in nutrient status, temperature, or salinity. This is consistent with the variations in the size of *A*. *ostenfeldii* cells in strains exposed to different experimental conditions [6, 16, 17, 59], which demonstrated that cell size variations are common in the life cycle of the species and are related to the culture growth phase and to the sexual vs. asexual origin of the cells. A larger mean cell volume during stationary phase as well as abundant large and small temporary cysts, most likely of asexual and sexual origin [70], were reported from Danish strain cultures by Østergaard and Moestrup [17]. Microscopic analysis of cells from our 12.5°C culture showed an abundance of large, dark, elongated cells suggestive of planozygotes. This culture condition also resulted in the largest numbers of double-walled (resting) cysts (unpublished data). Previous studies of the same strain showed that a temperature of 15°C results in significantly (*P* < 0.05; *n* = 90) larger cells than obtained at higher temperatures [6]. These observations suggest that sexual reproduction is induced in the cultures at low temperatures, which might also explain the fluctuations in toxin production observed at 12.5°C in this study as well as the higher error associated with the *G_m_*-parameter at low temperatures.

Little is known about the diversity, distribution, and production of GYMs by *A*. *ostenfeldii*. The only other producer of GYMs (GYM-A, GYM-B and GYM-C) identified to date is the phylogenetically distant dinoflagellate *Karenia selliformis* [11, 71–74], such that most studies of GYM toxin production have focused on this species. Thus far, the only study to quantify GYM production was conducted by Tatters et al. [75], using an isolate of *A*. *peruvianum* (syn. of *A*. *ostenfeldii*) from the New River Estuary, North Carolina (USA). Under nutrient-replete culture conditions, 12-methyl GYM concentrations peaked (up to 73.3 pg cell^-1^) during stationary phase (day 36), as was the case in most of our cultures. Harju et al. [8] provided a qualitative description of two separate GYM analogues produced by isolates from the Baltic Sea and Saanich (Canada) and of GYM-like compounds produced by some cultures established from the Baltic Sea. It is demonstrated that, as with SPX and PSP toxin, GYM production by *A*. *ostenfeldii* is geographically highly variable, including within a particular region [6, 13, 18, 29, 59, 76]. Thus far, lipophilic toxin production by *A*. *ostenfeldii* has been mainly linked to SPXs [34, 77, 78]. However, further studies quantifying GYM production in this species will provide greater insight into the dynamics of the biosynthesis of this toxin and its variability.

### Ecological implications

The accurate prediction and assessment of toxic episodes by dinoflagellates are hindered by the poor understanding of the factors affecting toxin production by dinoflagellates. Chemical, physical, and biotic factors are known to influence toxin production, including nutrients, temperature, salinity, irradiance, and grazing [68, 79]. For example, the complex ecological mechanisms underlying toxin production responses are evidenced by grazing, which can act to restrain dinoflagellate populations or enhance toxin content [80]. Our data are robust enough to allow the use of RSA as a predictor of a changing environment, although some limitations must be considered. First, the system giving rise to toxin production cannot be considered in its entirety. Second, because the results were derived from a single strain, any broader ecological interpretation must be made with caution [64]. According to our observations on the growth of the Baltic *A*. *ostenfeldii* strain AOTV-B4A, salinities in the range of 5–21 and temperature of 12.5–25.5°C are compatible with growth, although the respective values resulting in maximum growth were 11.2 and 25.5°C. Consequently, the strain appears to be a eurytherm adapted to brackish-water conditions. Although this strain may not be representative of all Baltic Sea populations of the species or of the species in general, for which we would need to work with many isolates, our data and those reported in the literature raise several interesting issues. For example, the intraspecific and intrapopulation variability of *A*. *ostenfeldii* with respect to temperature and salinity is not well known yet, although variations in the responses of different strains from different geographic locations characterized by a very wide range of environmental conditions have been extensively described [6, 17, 29, 34, 35, 58, 81]. Reaction norms of multiple isolates for temperature and salinity have not been published so far, but previous studies on Baltic isolates suggest that the environmental window described herein for *A*. *ostenfeldii* AOTV-B4A represents the range of Baltic Sea population(s) [16, 58]. An optimum of ca. 25°C is consistent with the findings of Kremp et al. [58] who reported general growth stimulation in response to an increased temperature, despite variability in the responses of eight strains of *A*. *ostenfeldii* isolated from the same site as our strain.

While further investigations into the variability of strain responses to salinity are needed, our results can be compared with those in the literature. Thus, the optimal salinities allowing growth as determined in the present work are in agreement with those reported by Kremp et al. [16] and Suikkanen et al. [29] for other isolates from the same region (Åland islands, between Finland and Sweden). However, in the strain from Limfjord, Denmark (Kattegat Sea) studied by Østergaard and Moestrup [17] as in the strain from Skagerrak studied by Suikkanen et al. [29], the values were higher and salinities of up to 35–40 were tolerated. This variation suggests that both Limfjord and Skagerrak strains belong to a different, high-salinity-adapted population, a conclusion supported by the genetic study carried out by Kremp et al. [13], who showed that strains from the Baltic Sea and Limfjord were grouped within distinct phylogenetic clades. The low optimum salinity (11.2) recorded for our *A*. *ostenfeldii* strain is consistent with its adaptation to the low salinities of the shallow coastal embayments of the northern Baltic Sea, where salinities are typically between 6 and 7 [16, 29]. Furthermore, our results along with those of previous studies [6, 29] show that a salinity > 25 is a limiting factor for growth. Extrapolated to the natural environment, this would limit the geographic distribution of the population to areas influenced by freshwater inputs, such as the most northern and eastern areas of the Baltic Sea [29]. The adaptation of *A*. *ostenfeldii* to specific environments of the Baltic Sea is indicative of an early post-glacial colonization and the continued isolation of the respective subpopulations due their limited dispersal [82].

Our results on the temperature preferences of one strain of *A*. *ostenfeldii* support the concerns expressed by other authors regarding the potential increase in blooms of this species as a possible response to climate change [58]. Summer conditions in the Baltic Sea, when the water is warm, stratified, and nutrient-poor are optimal for dinoflagellate growth [58] and for increased toxin production (19°C for PSP toxin and 20.9°C for GYM-A analogue). This is especially the case in shallow and sheltered embayments, where the temperature may be well above 20°C [16]. In fact, large summer blooms of *A*. *ostenfeldii* in the brackish estuaries and shallow coastal inlets of diverse areas during warm-water periods have become increasingly common in recent years [15, 33, 83].

## Conclusions

Both cell growth and net toxin (GYM and PSP toxin) production were directly responsive to temperature and salinity changes. While increasing temperatures stimulated growth as well as net toxin production, salinities higher than 21 were growth-limiting. The present study also provides the first quantitative determination of a GYM-A analogue in *A*. *ostenfeldii* and the first report of changes in its production in response to variations in temperature and salinity. The changes in PSP toxin and GYM-A analogue production suggest a mixed-growth-associated model, since net toxin production typically occurred in exponentially growing and in stationary-phase cells. The optimal temperature and salinity that resulted in maximum toxin concentrations were: 20.9°C and 17 for the GYM-A analogue and > 19°C and 15 for PSP toxins, respectively. The RSA presented herein is a valuable approach for evaluating the combined effect of temperature and salinity on *A*. *ostenfeldii* growth and net toxin production. While further studies estimating the magnitude of within-species variation are needed, our data suggest that warming of the water would stimulate both the growth of *A*. *ostenfeldii* and its toxin production.
