# Correction: A Kinetic and Factorial Approach to Study the Effects of Temperature and Salinity on Growth and Toxin Production by the Dinoflagellate *Alexandrium ostenfeldii* from the Baltic Sea

**DOI:** 10.1371/journal.pone.0147461

**Published:** 2016-01-25

**Authors:** The PLOS ONE Staff

There is an error in the PDF version of the published article. In the Material and Methods, under the subsection Experimental design and statistical analysis, the text should indicate the following:

$$F1=\text{Model }/\text{ Total error }F1\;\geq \;F_{d}{}_{en}^{num}$$

The Publisher apologizes for the error.
